# A Narrative Review of Visual Hallucinations in Migraine and Epilepsy: Similarities and Differences in Children and Adolescents

**DOI:** 10.3390/brainsci13040643

**Published:** 2023-04-10

**Authors:** Daniela D’Agnano, Salvatore Lo Cascio, Edvige Correnti, Vincenzo Raieli, Vittorio Sciruicchio

**Affiliations:** 1 Children Epilepsy and EEG Center, San Paolo Hospital, ASL Bari, 70132 Bari, Italy; 2 Child Neuropsychiatry Unit Department, Pro.MI.S.E. “G. D’Alessandro, University of Palermo, 90100 Palermo, Italy; 3 Child Neuropsychiatry Department, ISMEP, ARNAS Civico, 90100 Palermo, Italy

**Keywords:** headache, migraine, visual hallucinations, epilepsy, aura, children

## Abstract

Since the earliest descriptions of the simple visual hallucinations in migraine patients and in subjects suffering from occipital lobe epilepsy, several important issues have arisen in recognizing epileptic seizures of the occipital lobe, which often present with symptoms mimicking migraine. A detailed quantitative and qualitative clinical scrutiny of timing and characteristics of visual impairment can contribute to avoiding mistakes. Differential diagnosis, in children, might be challenging because of the partial clinical, therapeutic, and pathophysiological overlaps between the two diseases that often coexist. Ictal elementary visual hallucinations are defined by color, shape, size, location, movement, speed of appearance and duration, frequency, and associated symptoms and their progression. The evaluation of the distinctive clinical features of visual aura in migraine and visual hallucinations in occipital epilepsy could contribute to understanding the pathogenetic mechanisms of these two conditions. This paper aims to critically review the available scientific evidence on the main clinical criteria that address diagnosis, as well as similarities and differences in the pathophysiological mechanisms underlying the visual impairment in epilepsy and migraine.

## 1. Introduction

Migraine and epilepsy are two of the most common neurological disorders affecting pediatric neurological patients; they are potentially life-threatening, and lead to significant disability and reduction in quality of life in children and adolescents [1,2]. These two diseases are often comorbid in a non-coincidental way. Among epileptic patients, the prevalence of migraine ranges from 8 to 24% [1] and some authors [2] demonstrated an increased risk for unprovoked seizures in children having migraine with aura as opposed to migraine without aura (this finding is still to be confirmed [1]). Migraine and epilepsy share many clinical features: the episodic or recurrent occurrence of attacks, which are characterized by paroxysmal onset and distinct preictal, ictal, and post-ictal phases [3] with typical manifestations (changes in mood, behavior, and consciousness; changes in visual, motor, sensory, or speech functions) [4]. This phenotypic similarity alludes to shared mechanisms dealing with neuronal hyperexcitability arising from the cortex and modulated by subcortical connections. Despite some well-established differences in clinical features between pediatric migraine with aura and developmental epilepsies presenting with visual hallucinations (such as childhood occipital epilepsy and symptomatic childhood occipital epilepsies/Gastaut-type) [5,6], differential diagnosis may be challenging if atypical presentation occurs [7,8]. In this review, we report the primary evidence on the clinical tips that could help clinicians differentiate between epileptic and migraine visual symptoms.

## 2. Visual Symptoms in Migraine

Among children and adolescents suffering from migraine (5% in pre-pubertal children, with percentages increasing throughout adolescence), approximately one-third have migraine with aura (MA) [9,10,11]. Typical aura without headache is rarely described and actual prevalence is still debated; some authors report from 1.8 to 2% [12,13], and others found that 44% of patients are affected by migraine with aura [14]. The most common type of aura is visual aura (87.1%), followed by sensory (38.5%) and speech and language auras (15.6%). A confusional state is present in 10.9% of patients [10], whereas olfactory hallucinations accompany approximately 3.9% of pediatric migraine aura cases [15]. Panayiotopoulos described well the clinical features of visual hallucination in migraine. It is now well-known that they differ from epileptic hallucinations in terms of color, shape, size, location and movement in the visual field, frequency, and associated symptoms. A peculiar and distinctive feature of the visual aura is the gradual onset and progression, in contrast to the sudden onset typical of epilepsy, cerebral ischemia, or hemorrhage [7]. Based on their characteristics, visual symptoms can be distinguished as “negative” (vision loss) or “positive” (with flashing, shimmering, or scintillating patterns) [16]; and simple (dots or other simple shapes) or complex (more prominent and more elaborate vision disturbances, such as the perception of fortification spectra and other illusions). Usually, elementary visual disturbances in migraine aura develop slowly within 4 min and usually last 15–20 min, and up to 60 min (IHS 2018); they have mainly uncolored or black and white patterns, although frequency of up to 40% of colorful aura is reported [17,18], and are represented by linear or zig-zag achromatic shapes [18,19], first appearing in the center of the visual field and then moving peripherally. In most cases, simple positive visual hallucinations are followed by a scotoma. Scintillating scotoma and blurry vision are the most frequent visual symptoms in children, followed by tunnel vision and zig-zag lines [20]. Moreover, most frequently than adults, children complain of color dysgnosia caused by unusual color brightness that prevents patients from recognizing color shades [20,21]; less frequently, adult patients complain of dimmed colors or achromatopsia, usually often associated with both prosopagnosia and loss of spatial orientation [22]. Theoretically, color perception changes could result from photophobia or a direct migraine effect on color processing, as Noseda et al. suggested, originating in the retina and thalamus rather than in cortical visual processing areas [23]. Visual aura rarely occurs with accompanying neurologic findings that are typical manifestations of epileptic seizures (i.e., tonic deviation of the eyes and alterations in consciousness). In our digression, it is essential to make a point: headaches are not always associated with auras and, in particular, auras do not always precede a headache. In this regard, we recall an uncommon diagnosis of typical aura without headache (TAH), which is neither accompanied nor followed by headache [24].

## 3. Visual Symptoms in Epilepsy

In most pediatric cases, visual disturbances are the typical manifestation of idiopathic childhood epilepsy with occipital paroxysms (Gastaut-type). This idiopathic epilepsy represents approximately 2 to 7% of benign childhood focal seizures [6], with an estimated prevalence of 0.3% in children and peak incidence between 8 and 9 years of age [6]. The hallmark of this childhood epilepsy syndrome is predominantly diurnal visual seizures [6] characterized by brief elementary visual hallucinations, deviation of the eyes, and blindness. Sometimes, autonomic symptoms follow the visual symptoms and evolve into a dyscognitive seizure. Usually, but not always, typical occipital paroxysms are detectable using EEG [25].

Elementary visual hallucinations are stereotyped, multi-colored, and circular. Frequently, they appear on the edge of a temporal hemifield, multiply in number and size, often move horizontally towards the other side, and may flash or be static. Usually, they develop within seconds and generally last up to 1 to 3 min. In a recent comparative analysis of visual symptoms, the median duration was reported as 56 s and 20 min in epileptic and adult patients affected by migraine, respectively [26]. Stereotypic lateralization of the visual phenomena was reported to be significantly more common in epilepsy than in migraine [26]. Visual hallucinations can occur in different diseases and might be caused by structural lesions in the posterior temporal–occipital regions, including cortical dysplasia, encephalomalacia, low-grade glial tumors, vascular malformations, and occipital calcifications [27].

## 4. Visual Symptoms in Both Conditions: Epilepsy and Migraine

**Figure 1:**
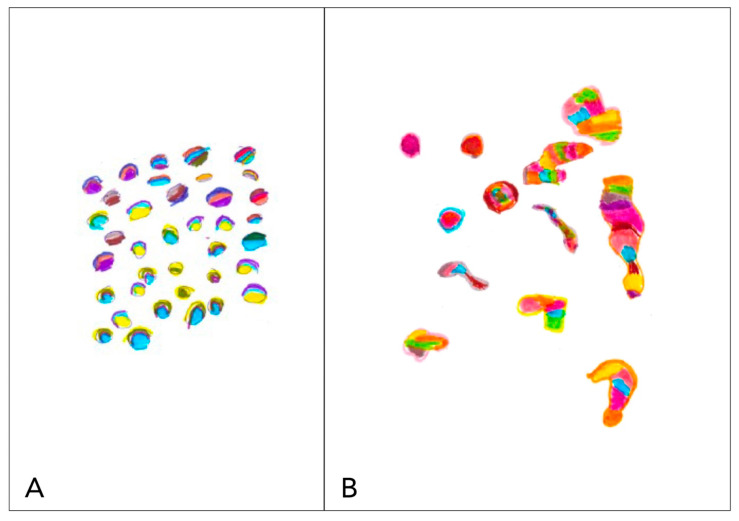
Graphical representation of phosphenes. We report two explanatory drawings, drawn by two children who arrived at the Department of Neuropsychiatry of Di Cristina Hospital in Palermo. (**A**) Graphic representation of phosphenes in a child with occipital epilepsy with seizure without loss of consciousness. (**B**) Graphic representation of phosphenes in a child with migraine with aura. The high similarity between the two symptoms complicates the differential diagnosis. (Courtesy of Dr. V. Raieli).

There is substantial variability in the characteristics of visual aura, with some commonalities across individuals. Recently, Viana et al. undertook a comprehensive review of all currently available data from clinical studies dealing with visual hallucinations in migraineur adults [28] and showed how different, and often complex, visual disturbances could be. Despite the smaller amount of evidence compared to adult patients, unusual visual aura has also been reported in children and adolescents. Polychromatic figures and formed shapes (e.g., dots, circles, triangles, squares, stars) [29] can mimic occipital seizures but duration, localization, and movement pattern, as previously mentioned, may provide guidance for the correct diagnosis (Figure 1).

Unfortunately, visual disturbances such as visual loss (scotoma, amaurosis, and blindness), palinopsia, blurring vision, and macro or micropsia (as in the Alice in Wonderland syndrome) are more challenging and may mislead clinicians. In those cases, a detailed clinical history may reveal the event’s true nature and further direct investigations [30].

### 4.1. Visual Loss

**Figure 2:**
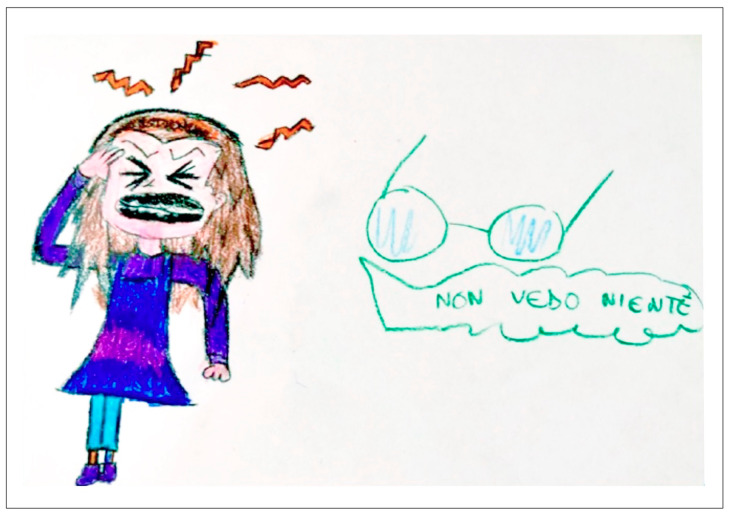
Graphical representation of blindness. We report explanatory drawing of blindness, drawn by a child with migraine who visited the Department of Neuropsychiatry of Di Cristina Hospital in Palermo. Translation of the written portion: “I don’t see anything”. (Courtesy of Dr. V. Raieli).

Ictal amaurosis, blindness, or severe blurring of vision, limited to one hemifield, quadrant, or involving the entire visual field are common ictal phenomenon in idiopathic childhood occipital epilepsy (Gastaut-type), occurring in-two thirds of such patients and usually following the visual hallucinations, and last up to 5 min [31]. However, they may occasionally constitute the first symptom or the only ictal manifestation with abrupt onset [32,33,34]. In 70% of patients [4], it is associated with horizontal deviation of the eyes and progresses to varying associated focal convulsions. In the absence of any other seizure phenomenon, it may not be possible to differentiate migraine-induced transient cortical blindness from that induced by a seizure disorder. Migraine-induced transient cortical blindness can result from the scotoma resulting from the visual aura or can underlie a basilar or retinal migraine (Figure 2).

Retinal migraine is sporadic among pediatric migraine patients, accounting for <2%; in this disorder, monocular scotoma or vision loss is reported, rather than hemifield deficits typical in the visual aura.

### 4.2. Palinopsia and Polioplia

Palinopsia is derived from the Greek words palin (again) and opsis (vision) and describes the perseveration of visual images. Palinopsia is a cardinal symptom of ictal clinical manifestations of occipital lobe seizures [34]. Belcastro et al. 2011 [35] described palinopsia events that lasted for seconds in 10% of 200 adults affected by migraine with and without aura. Polyopia is an uncommon visual phenomenon and an infrequent symptom of central nervous system disease, mainly occipital or temporal lesions [36], often associated with palinopsia, defined as the perception of many copies of objects or faces [37]. An interesting case report with a child affected by polioplia associated with the hemianopsia during migraine attack has been described in children affected by migraine [38,39]

### 4.3. Visual Snow

Visual snow is described as the electronic “snow” on a television set when the primary video signal is either reduced or absent, as well as pixelated fuzz and bubbles. It can be due to different etiologies and it has variable duration. When this symptom is associated with palinopsia, enhanced entoptic imagery, “nyctalopia”, and photophobia/photosensitivity, visual snow syndrome (VSS) can be diagnosed. In the anecdotal cases of migrainous patients reported in the literature [40], visual snow is usually short and without associated symptoms. In contrast, VSS was reported by Polster et al. [41] in two patients with suspected occipital epilepsy.

### 4.4. Other Complex Positive Symptoms

In migraineur children, visual hallucinations including landscapes, animals, human bodies, or faces, or the perception of mysterious objects that may aggregate to form complex scenes, are limited to anecdotical reports [42,43]. As epileptic symptoms, Panayiotopoulos described only three patients who reported “large objects, probably people, which I cannot identify”. Clear perception of people is reported in adults affected by different disease (hemorrhage, stroke, malformations, neoplasia) and are usually associated with medial temporolimbic seizure [44], and less commonly with lateral temporal seizure and rarely with occipital seizure [45]. In contrast, perceptual distortions of the patient’s body or objects in the environment, as described in Alice in Wonderland syndrome (AWS), are frequently reported in children [12,46]. AWS can present as micropsia (objects appear too small), macropsia (objects appear too large), metamorphopsia (objects appear too fat, thin, short, tall, etc.), teleopsia (objects appear further away than they are), and pelopsia (objects appear closer than they are). The symptom duration is widely variable, differs in each person, and resolves without sequelae. The cause of these symptoms is debated and remains unknown in almost 20% of cases [46]. In 2016, Blom et al. [46] showed that out of 166 published cases of AIWS, migraine was the most common cause (27.1%), followed by infections (22.9%), principally EBV (15.7%), brain lesions (7.8%), medication (6%) and drugs (6%), psychiatric disorders (3.6%), temporal or focal epilepsy (3%), disease of the peripheral nervous system (1.2%), and others (3%). In nearly 50% of patients [47], a family history of migraine or Alice in Wonderland syndrome can be detected. When occurring in children without other conditions, it usually predicts a higher chance of developing migraines in the future [16]. When AWS is associated with migraine, the symptoms can occur before, during, or after a migraine headache; micropsia and teleopsia have been more frequently reported [48].

## 5. Anatomy of Visual System

The visual system is an intricate neuronal structure that transports information from the retina to the visual cortex. The retina is the first nerve portion of the visual pathway, consisting of 10 cell layers, of which the first is the most important and consists of the receptors (cones and rods) that synapse with the ganglion cells of the last layer [49]. The axons of the retinal ganglion cells pierce the sclera to assemble the optic nerves that pass via optic canals of the sphenoid bone to the middle cranial fossa and decussate to form the optic chiasm in a point anterior to the pituitary infundibulum [50]. By dividing the visual field into quadrants, we know that a decussation (crossing) of optic fibers occurs at the chiasm level: the nasal fibers of one eye cross and join the temporal optic fibers of the contralateral eye. The optic tracts continue postero-laterally, passing around and behind the tuber cinereum and anterior perforated substance and around the cerebral peduncles to terminate in the lateral geniculate nuclei of the thalamus [51]. The superior colliculi and the midbrain pretectum receive important innervations from the optic tracts, respectively associated with saccadic eye movements and pupillary reflexes. The lateral geniculate nucleus is comprised of six layers of cell bodies. The optic tract fibers arrive there with a specific and complex subdivision that is based not only on the retinal spatial origin (nasal retinal fibers to layers 1, 4, and 6 and the temporal to layers 2, 3, and 5) but also based on a functional subdivision (arising from the so-called M and P cells, which have specific functions with respect to acquisition of movements or chromatic contrast) [52]. Fibers originating from the lateral geniculate bodies form optic radiations (or geniculo-calcarinic tracts) that pass through the retrolenticular portions of the inner capsules and travel to the visual cortex, above and below the calcarine fissures. The most caudal part of the primary visual cortex receives the innervation from the fovea, while more rostral portions of the cortex receive the innervation from the increasingly peripheral regions of the visual field. Each hemisphere represents the projection of the contralateral visual field; the superior portion of the calcarine fissure corresponds to the inferior visual field, while the inferior portion corresponds to the superior field. Finally, as is often the case in the cerebral cortex, multiple inter-cortical connection pathways exist in the connections with the centro-medial region of the occipital lobe, whose lesion results in prosopagnosia, quadrantanopia, and topographagnosia; superior longitudinal fasciculus, the inferior fronto-occipital fasciculus, and the inferior longitudinal fasciculus make these connections with parietal and frontal regions.

## 6. Pathophysiology: Neocortical Dysexcitability

The pathophysiology mechanism involved in visual symptoms in migraine aura is yet to be fully clarified, the anatomical locus from which the visual symptoms spreads is still unclear, and the visual percept that aura produces remains uncertain.

In 2013, Hansen et al. [53] reported detailed maps of the visual perception of hundreds of migraine auras of a patient over a period of 18 years. The drawings analysis revealed that despite distinct regions of the occipital cortex having a higher propensity to initiate an aura, there is not a hyperexcitable focus in the visual cortex but there can be multiple distinct sites of aura initiation that spread non-concentrically in the occipital cortex with a variable extent of propagation.

Cortical spreading depression (CSD) phenomenon is generally assumed to be the mechanism underlying aura and one of the pathophysiological links between visual symptoms in occipital epilepsies and visual aura in migraine. CSD is a neuronal and glial electrophysiological phenomenon, accompanied by alterations in ionic concentration, cerebral blood flow, variation in neurotransmitter level, and the brain [54,55], that differs from spreading depolarization; CSD is characterized as short excitation, followed by long-lasting depression of cortical activity that originates from the occipital lobes of the brain, with a postero-anterior course with occipital origin and vanishing of the central sulcus. Functional magnetic resonance imaging data demonstrate blood oxygenation level dependent signal changes over the visual cortex during visual aura. Perfusion studies show the alterations in cortical blood flow represented by initial hyperemia followed by sustained oligemia, which restores homeostasis. A particular sensitivity to cortical spreading depression and uniform decreases in blood flow in the occipital lobe is observed; this may explain the clinical observation that about 90% of patients suffering from migraine with aura show visual symptoms during aura. Nevertheless, the cortical spreading depression (CSD) phenomenon is poorly adaptable to many different, often complex, visual disturbances in visual aura [28]. Several hypotetic cellular mechanisms, including channelopathies, impairment of ion homeostasis in neuronal and glial cells, disturbance of GABA-ergic or glutamatergic systems, and disorders of mitochondrial function, are supposed to underly the disturbances. Molecular mechanisms leading to visual hallucination appearance are thought to involve increased serotonin receptor binding ability in the visual pathway [43]. Raieli et al. [7] recently discussed clinical aspects of pediatric atypical auras, which seem more challenging to frame with the mechanisms of cortical spreading depression. Based on the possibility of cortical–subcortical onset of local areas of “spreading depression” with a possible role of the thalamus, they suggest the presence of multiple, synchronous and asynchronous, cortical and subcortical spreading depression networks.

In order to explain how the electrical phenomenon is triggered, several hypotheses have been made: Harreveld [56] and Grafstein [57], supposed a role of various conditions such as hypoxia, ischemia, and hypoglycemic activity [55]. A recent review collates most of the triggering factors that make one prone to the CSD condition, listed below [58]:

- Inadequate tissue oxygenation;
- Fluctuations in ionic concentration (potassium, calcium, sodium, chloride);
- Change in direct current potential;
- Cytotoxic edema, mediated by higher osmolality (more significant influx of sodium and calcium);
- Dendritic beading with spine loss;
- Alteration in glucose metabolism;
- Lactic acid accumulation;
- Decreases in pH and ATP with increased cAMP and ADP levels;
- Disregulation of release and diffusion of serotonin, neurokinin, calcitonin gene-related peptide, and brain-derived neurotrophic factor.

In idiopathic childhood occipital epilepsy (Gastaut-type), the interictal EEG shows high-amplitude occipital spikes often associated with a background with bilateral synchronous and asynchronous focal slowing. This electric layout suggests a calcarine/occipital hyper-excitability. During an ictal event, the fast rhythmical spikes that characterize the EEG-graphic data of seizure are the mark of the cortical depolarization, leading to the visual symptoms. Another point to consider is the “hemicrania epileptica” concept, coinciding with EEG epileptic activity localized homolaterally to migraine pain, probably associated with an increased cerebral blood flow observed during the pre-ictal or ictal period, which triggers trigeminovascular activation, resulting in headache [59].

## 7. Final Consideration

Visual aura in migraine and visual seizures are distinguishable when all their typical features and associated or following symptoms are present and collected by detailed clinical history. The best predictive parameters for a migraine visual aura are the presence of accompanying symptoms, such as nausea, vomiting, photophobia or phonophobia, and, obviously, headache, which are long-lasting. In particular, a duration of more than 5 min was found to exclude epilepsy aura (median duration of 56 s) in favor of migraine aura (median duration of 20 min) with sensitivity of 100% and specificity of 92% [26]. A detailed quantitative and qualitative clinical scrutiny of the timing, progression, and characteristics of visual impairment can contribute to avoiding mistakes. Nevertheless, a minority of migraine children shows a shorter aura (lasting <5 min), and atypical clinical features. In addition, rarely, but probably in an underestimated percentage of cases, visual aura can arise without headache; in this case, differential diagnosis may be challenging. Uncolored or black-and-white patterns, such as linear or zig-zag achromatic shapes, moving peripherally in the visual field, or scintillating scotoma and blurry vision, are the most frequent visual symptoms reported in children affected by visual aura. Unusual colors, brightness, short-lasting visual snow, micropsia, and teleopsia can less frequently underlie a visual aura. Palinopsia, visual snow syndrome, Alice in Wonderland syndrome (with predominant macropsia, metamorphopsia, and pelopsia), or complex visual hallucinations (in the shape of a body, landscape, or animal) are very rarely visual features of aura and evoke other etiologies (Table 1).

Although it is beyond the scope of this review, when dealing with the burden between epilepsy and migraine it is mandatory to underline that epilepsy is not infrequently accompanied by preictal, ictal, and post-ictal headaches that often have migraine features [66,67,68]. In particular, the association between epilepsy and migraineur post-ictal headache (PIH) is interesting since they are paroxysmal and chronic, and both often respond to antiepileptic drugs. However, their specific pathophysiological mechanisms are not well-known [66].

Unfortunately, definitions of epilepsy-related headaches differ across most of the studies and constantly change in the International Classification of Headache Disorders, which adds difficulties and confusion in identifying this condition by neurologists and researchers; as a result, it is undertreated and misunderstood [69,70]. In 2012, an Italian study even suggested the new concept that the “ictal epileptic headache” should be classified as an “autonomic seizure” [69]. By comparison, according to ICHD-III, post-ictal headaches occur within 3 h of an epileptic seizure and resolve within 72 h of seizure end. Few studies on aspects of PIH are available in the literature; however, PIH is a frequent condition reported by almost half of the patients, usually as migraine headache, which seems to occur more commonly after generalized seizures than after partial seizures, except for occipital lobe epilepsy [68,71]. In fact, as shown in a multilevel linear modeling study involving 302 subjects, the factors that most influenced the occurrence of PIH are a family history of migraine, drug-resistant epilepsy, and subjects generalized seizure onset type. Other [72] associations with PIH reported by previous authors include a younger age of onset of epilepsy [71]. These data are confirmed in the pediatric population. An Italian cross-sectional multi-center study of peri-ictal and inter-ictal headache in children and adolescents with idiopathic epilepsy found an even higher PIH incidence of 62%. They also found that within the group of 81 patients with PIH, 61 (75.3%) had migraine without aura features and 5 (6.1%) had migraine with aura features. Another point [73] to consider, dealing with the link between headache and epilepsy, is that migraine aura and headaches may trigger epileptic seizures. William Lennox first used the term “Migralepsy” to describe a syndrome of migraine with aura where the migraine is almost immediately followed by an epileptic seizure in a way that gives rise to the suspicion that the one triggered the other [74,75].

## 8. Conclusions and Future Direction

Migraine and epilepsy are distinct neurological diseases with specific clinical features and underlying pathophysiological mechanisms. However, numerous studies have highlighted the complex and multifaceted relationships between the two conditions.

Nevertheless, currently, there is no clear pathophysiological explanation for the marked heterogeneity of visual symptoms and no biomarker for disease susceptibility.

The overlap of features between visual aura and visual seizures can make differential diagnosis challenging but mistakes can be avoided by collecting a detailed clinical history. For this reason, more reports of accurate descriptions of the clinical features of visual aura in children and adolescents are needed to better define symptoms and distinctive features in both conditions. Deep knowledge of pathophysiology mechanisms involved in cortical excitatory-inhibitory balance, promotion of hyper-reactivity to pain, and advancements in comprehending the potential neural mechanisms linked with migraine are necessary to understand and provide specific treatment options [76]. Some interesting data belong to the diffusion-weighted imaging (DWI) sequences, based on the difference in magnitude of water diffusion. These have improved our knowledge of headache disorders by detecting brain microstructural alterations in patients with migraine headaches [77]. However, longitudinal studies will be beneficial in investigating the development of microstructural abnormalities during disease or treatment. A recent study showed how the use of machine learning in a multivariate model, based on clinical and structural data, may improve the classifier’s ability to distinguish individuals with migraine from those with persistent post-traumatic headache [78]. Today, despite the most advanced methods of functional exploration and data collection and analysis, the diagnosis of childhood headache and epilepsy, based on aura symptoms, still remains a stimulating challenge for clinicians. Combined investigations, such as neuroimaging and functional studies, are needed to provide a better understanding of the underlying pathophysiological mechanisms to avoid diagnostic mistakes.

## Figures and Tables

**Table 1:** Characteristics of visual symptoms: differential diagnosis between migraine and epilepsy.

Symptoms			Migraine	Epilepsy	ref
Negative		Scotoma Monocular Binocular	Frequent Rare		Schwartz et al. [60]
		Blurred vision	Frequent	Rare	Petrusic et al. [20]
		“Tunnel” vision	Frequently reported in children	Anecdotal in adults	Petrusic et al. [20] Christian et al. [61]
		Blindness Monocular Binocular	Consequence of scotoma; very rare occurrence in adults; female-predominance	2/3 of patients	Kosnik et al. [62] Caraballo et al. [6] Aldrich et al. [63] Queiroz et al. [17] Rozen et al. [64]
Positive	Simple	Bright light/unformed flashes of light/ star-shaped figures	Commonly reported; a centripetal/- fugal drift of the phosphenes	Horizontal drift stereotypic lateralization	Hartl et al. [26]
		Scintillating scotoma	Frequent	Rare	Petrusic et al. [20] Panyiotopoulos et al. [5]
		Zig-zag or jagged lines	Frequent		Petrusic et al. [20] Russel et al. [18] Panyiotopoulos et al. [5]
		Black dots	A centripetal/- fugal drift of the phosphenes; relatively common	Horizontal drift stereotypic lateralization	Hartl et al. [26] Queiroz et al. [17]
		Color dysgnosia Color brightness	Frequent	No data	Petrusic et al. [20] Hadjikhani et al. [21]
		Phosphenes (small bright dots)	A centripetal/- fugal drift of the phosphenes	Horizontal drift stereotypic lateralization	Hartl et al. [26]
		Curved or circular lines	Relatively common		Queiroz et al. [17]
		“Bean-like” forms like a crescent or C-shaped	Frequent		Queiroz et al. [17]
		Flickering light	A centripetal/- fugal drift of the phosphenes; Frequent		Russel et al. [18]
		Palinopsia	No report in children	Frequent	Adcock et al. [34]
		Polyopia	One report	rare	Raieli et al. [38] Kataoka et al. [36]
	Complex	Visual snow	Seven anecdotal cases; isolated, short-lasting	Two reports; visual snow syndrome	Simpson et al. [40] Polster et al. [41]
		Mosaic vision	Relatively common		Podoll et al. [65] Queiroz et al. [17]
		Fractured Vision	Relatively common		Podoll et al. [65]
		Corona effect	Single or two extra contours, surrounding parts or the complete contours of an object, in black/white or in color, around perceived or illusory images	Not described	Podoll et al. [65]
		Complex hallucinations	Rare	Frequent	
		Alice in Wonderland syndrome (micropsia, macropsia, teleopsia, metamorphopsia, pelopsia)	When AWS is associated with migraine, micropsia and teleopsia have most frequently been reported	Rare	Shevell et al. [12] Blom et al. [46]
		People, animals, landscape	Extremely rare	Rare	Akiyama et al. [43] Smith et al. [42]

## Data Availability

No new data were created, and clinical data are unavailable due to privacy or ethical restrictions.
